# Cough up for just a cup of coffee: Pharmacoeconomics of depression

**DOI:** 10.4103/0019-5545.39751

**Published:** 2008

**Authors:** G. Swaminath

**Affiliations:** Department of Psychiatry, Dr. BR Ambedkar Medical College, Kadugondanahalli, Bangalore, Karnataka, India

Unlike psychotics who thrust themselves into the priority list, benign depressives rarely get precedence in management. The alacrity and ease with which other medical professionals refer psychotics and other restless patients to psychiatrists is not seen when they deal with depressives. Depression is a high-prevalence disorder with moderate-to-severe intensity and accounts for one-third of cases visiting primary healthcare clinics. In the community, depression remits and recurs, and the frequency of remission may lead clinicians to underestimate the probability of relapse.[1]

Depression is a chronic, relapsing, recurrent disorder, the fourth most important determinant of the global burden of disease, and the largest determinant of disability in the world. As patients do not seek treatment and when they do, efficacious treatments are not always used effectively, there is little hope of reducing this burden.[1]

Comparing depression with other illnesses with high burden, such as coronary heart disease (CAD), cancer and AIDS, shows interesting patterns. The high-profile AIDS has a low lifetime prevalence of 0.2, compared to depression with a prevalence of 15 and the other two with a lifetime prevalence of around 6-7. A majority of people suffering from depression (more women) and AIDS (more men) are in their 20s to 40s, while CAD and cancer patients (equal men and women) are usually beyond the age of 45. CAD and cancer have a high recognition rate, variable treatability and survival rates, while AIDS has a high recognition rate, low treatability and low survival rate. In contrast, depression has a poor recognition rate, but excellent treatability and excellent survival rate with adequate treatment. In addition, the cost per year of treating depression is very low as compared to the other illnesses.

In evaluating the economic cost of depression, the cost of intervention, namely the cost of detection, treatment, prevention, rehabilitation and long-term care have to be weighed against non-intervention [related to morbidity and mortality and loss of productivity of patients].

Among the direct costs, hospital and outpatient expenses form the bulk. Co-morbid illnesses corner a formidable part of the costs.[2] Considerable funds would be spent on work-ups and investigations in pursuit of a ‘physical diagnosis’. Over-utilizers of medical care services have a high incidence (68%) of lifetime prevalence of depression.[3] Surprisingly, drugs constitute just 6% of the direct costs of depression and 1% of the overall expenditure.

Efficacy, side-effects and factors influencing compliance among different antidepressant drugs determine the choice of drug. There have been reviews recommending SSRIs over older antidepressants, despite the cost. Lesser side-effects and good compliance are among the reasons cited in the western literature.[4]

However, in India, the cost factor is not a major issue. Thanks to some highly criticized policies such as the drug price control order (DPCO) and the policies on patents (product patent vs process patent), psychiatric drugs have been priced 75-90% cheaper than prices obtaining in the developed world. As an example, the cost of carbamazepine has fallen drastically from the time of its introduction two decades ago. The shift to product patents three years back has not made a change as, only drugs which are out of the patent list have been introduced.

Antidepressants have become cheaper over time. The recent price reduction of bupropion (sustained release form) by 60% resulted in the doubling of its sales and recovery of costs. Barring paroxetine, mirtazapine, duloxetine and tianeptine, the average per-day adult dose of antidepressant costs less than Rs. 8. Sertraline and escitalopram, though newer drugs, cost less than the average daily doses of tricyclic antidepressants in India, unlike that in the West.

In conclusion, depression is a chronic, relapsing but treatable disorder, which disables patients in their highly productive years. The price of depression is essentially in not being detected and treated. The depressed patient, to overcome his suffering, needs to take drugs priced just as much as a cup of hot coffee; coffee, not in an exclusive place, but a modest joint of the kind most middle-class Indians frequent! Given this scenario, early detection and proper management with proactive follow-up could reduce the socioeconomic burden of depression and help rationalize healthcare rather than ration it.
